# A Systematic Review of Behaviour Change Techniques within Interventions to Increase Vaccine Uptake among Ethnic Minority Populations

**DOI:** 10.3390/vaccines11071259

**Published:** 2023-07-19

**Authors:** Winifred Ekezie, Aaisha Connor, Emma Gibson, Kamlesh Khunti, Atiya Kamal

**Affiliations:** 1 Diabetes Research Centre, University of Leicester, Leicester LE5 4PW, UK; 2 Centre for Ethnic Health Research, University of Leicester, Leicester LE5 4PW, UK; 3 School of Social Sciences, Birmingham City University, Birmingham B4 7BD, UK

**Keywords:** vaccine hesitancy, vaccine uptake, ethnic minorities, barriers, facilitators, interventions, systematic review, behaviour change techniques, minoritized groups

## Abstract

COVID-19 caused significant morbidity and mortality amongst ethnic minority groups, but vaccine uptake remained lower than non-minoritised groups. Interventions to increase vaccine uptake among ethnic minority communities are crucial. This systematic review synthesises and evaluates behaviour change techniques (BCTs) in interventions to increase vaccination uptake in ethnic minority populations. We searched five databases and grey literature sources. From 7637 records identified, 23 studies were included in the review. Interventions were categorised using the Behaviour Change Wheel (BCW) and Behaviour Change Taxonomy v1. Vaccines included influenza, pertussis, tetanus, diphtheria, meningitis and hepatitis. Interventions were primarily delivered in health centres/clinics and community settings. Six BCW intervention functions and policy categories and 26 BCTs were identified. The main intervention functions used were education, persuasion and enablement. Overall, effective interventions had multi-components and were tailored to specific populations. No strong evidence was observed to recommend specific interventions, but raising awareness and involvement of community organisations was associated with positive effects. Several strategies are used to increase vaccine uptake among ethnic minority communities; however, these do not address all issues related to low vaccine acceptance. There is a strong need for an increased understanding of addressing vaccine hesitancy among ethnic minority groups.

## 1. Introduction

People from ethnic minorities were disproportionately affected by COVID-19, with an increased risk of severe infection and worse clinical outcomes than White individuals, including disproportionately higher cases and deaths [1]. However, certain ethnicities and minority populations in the western context have been more reluctant than others to receive the COVID-19 vaccine [2,3,4,5]. In the United Kingdom, people from Black, Pakistani and Bangladeshi communities had lower vaccine uptake despite being among the high-risk groups [2,6,7]. In 2021, it was reported that in Great Britain, Black or Black British adults had the highest rates of COVID-19 vaccine hesitancy (18%), followed by those from a South Asian background (17%), compared with White adults (4%) [8,9]. Similar variability in COVID-19 vaccine acceptance rates has been reported in different countries, with low acceptance rates being more pronounced in the Middle East, Eastern Europe and Russia, and higher acceptance rates in East and South East Asia [10]. Low vaccine uptake amongst some ethnic minority groups existed before the COVID-19 pandemic and has been associated with lower levels of vaccine confidence, uptake of routine vaccines and trust in vaccine services, all of which have been reflected in the COVID-19 vaccine uptake rates [11,12,13].

Vaccine hesitancy, the delay in acceptance or refusal of vaccination despite the availability of vaccination services, poses a threat to controlling COVID-19 and other vaccine-preventable diseases; hence, the World Health Organization (WHO) considers it one of the top 10 global health threats [14]. Changing behaviour is complex, and therefore, a systematic approach is required to understand factors influencing vaccine uptake, such as knowledge, beliefs, attitudes and behaviours of the targeted population group [15]. It is important to address systemic (access to health services and information, the structure and strength of the local healthcare system and service funding), individual (perceived efficacy of vaccines, risk perception, health literacy) and social barriers (social support and networks) to vaccine uptake [16,17]. Vaccine hesitancy can be reduced, and uptake increased when interventions target emotional, cognitive and social determinants that can either hinder or facilitate this behaviour through culturally appropriate information and messaging, policy and vaccine delivery [18,19]. Successful immunisation programmes generally result in high vaccine effectiveness and adequate uptake of vaccines.

A key priority in addressing barriers to vaccine uptake is identifying ways to engage with and deliver vaccinations to ethnic minorities and other vulnerable population groups. More targeted vaccination interventions are essential, as this can help understand and address concerns and barriers to uptake among specific groups, and this is beneficial for achieving national vaccination targets [20]. Identifying the barriers and facilitators to vaccine uptake and existing intervention behavioural strategies that have been effective among ethnic minorities could help address vaccine hesitancy in these groups [21,22].

Evidence of interventions to increase vaccination uptake exists, but evidence of vaccination interventions by ethnicity and race and the effectiveness of the interventions in increasing vaccine uptake is limited [23]. Studies that have explored vaccine access and uptake among ethnic minority groups often do not highlight behavioural components within interventions that influence uptake [11,24,25,26]. This limits our understanding of the mechanism of change resulting from interventions and the identification of how intervention content can be modified to increase vaccine uptake by drawing on specific behaviour change techniques designed to address barriers and facilitators of vaccine uptake among ethnic minority groups [21].

**Figure 1:**
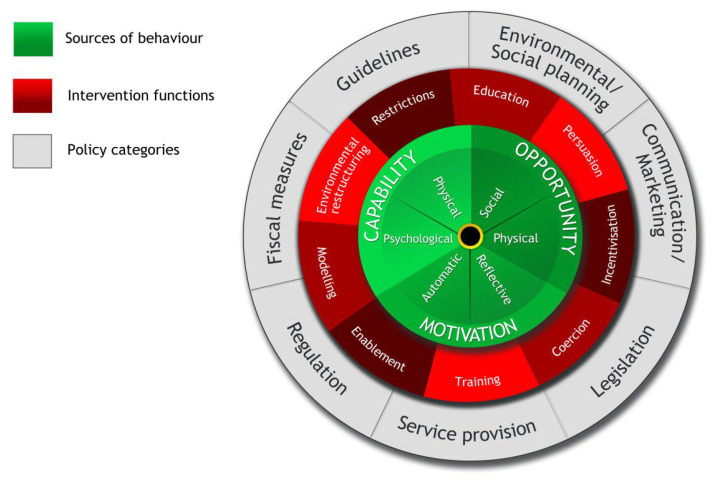
The Behaviour Change Wheel [32].

Interventions based on principles drawn from evidence and theories of behaviour and behaviour change have shown to be more effective [15]. Intervention development guidelines recommend using evidence-based behaviour change strategies [27], which requires understanding the behavioural elements of particular interventions that would improve vaccine uptake. Consideration is needed for the targeted population in relation to factors underlying vaccination uptake behaviour [28]. Behavioural determinants vary for different ethnic and racial minority groups, so it is important to identify and target these influences [29]. In 2000, the Medical Research Council (MRC) published a framework to help researchers to develop and evaluate complex interventions [30]. These guidelines outlined the importance of developing a theoretical understanding of causal mechanisms of action within interventions by drawing on existing evidence and theory [31]. In addition, the Behaviour Change Wheel (BCW) and Behaviour Change Technique Taxonomy v1 (BCTTv1) are comprehensive tools for identifying and describing specific behavioural components useful for intervention content [32,33]. The BCW is a synthesis of 19 frameworks of behaviour change that can be used to characterise intervention components that contribute to behaviour change; within the BCW is the inner hub (capability, opportunity, and motivation, i.e., COM-B model), which outlines sources of behaviour that could be the target for interventions [32]. (Figure 1) While the BCTTv1 is a 93-item taxonomy of behaviour change techniques (BCTs) [33], (Supplementary Table S1) the two frameworks are complementary and have been used to identify behavioural components in public health interventions, clinical trials and previous systematic reviews [34,35,36,37,38].

As some racial and ethnic minority communities have lower vaccine uptake, which was evident during the COVID-19 pandemic, it is essential to identify interventions and strategies that can improve vaccine uptake and reduce hesitancy among diverse populations and how this could inform the development or modification of interventions to support COVID-19 vaccination programmes in ethnic minority communities. Many different types of vaccinations are available, but in the current study, only vaccines that work against similar infections to COVID-19 (excluding COVID-19 vaccines) were reviewed to extrapolate learning from previous interventions to inform future COVID-19 and similar vaccine programme interventions.

### Review Questions

This systematic review sought to answer the following questions:

1. What intervention strategies, targeted at people from racial and ethnic minority backgrounds, can increase vaccination uptake?

2. What BCTs are included in interventions designed to increase vaccine uptake in racial and ethnic minority populations?

## 2. Methodology

This systematic review followed the Preferred Reporting Items for Systematic Reviews and Meta-Analyses (PRISMA) guidelines [39]. The protocol was pre-registered on the International Prospective Register of Systematic Reviews (PROSPERO ID: CRD42021239010) [40].

### 2.1. Search Strategy and Selection Criteria

Initial scoping review searches of three databases (PubMed, CINAHL and PsycInfo) and registered PROSPERO protocols indicated there were no published reviews or protocols in this area. As a result, a full systematic review of the literature was undertaken with searches for published and unpublished studies.

The search strategy was applied to the following databases: MEDLINE, EMBASE, CINAHL, EBSCOhost, and PsycInfo, hand searching was conducted over the last six years in two key journals (Vaccine and Vaccines). These databases and journals were selected based on their coverage of public health and vaccination topics. For the grey literature, a search was conducted through the first 10 pages of Google Scholar and pre-print databases (SocArXiv, MedRXiv, PsyRXiv, and SSRN). The search terms were based on a combination of keywords for three key concepts: “vaccine hesitancy” AND “minority ethnic groups” AND “intervention”. Within each concept, keywords were combined with Boolean search operators. Table 1 shows the keywords included in the search strategy for each concept. The asterisk * symbol was used as a truncation at the end of root words to broaden the search terms so it captures and includes various word endings and spellings.

Searches of databases had no date restriction and included papers from inception to 24 March 2021, and the search was updated 27 June 2022. Searches of pre-print databases were conducted and included papers up to 21 July 2022, after the last searches were conducted. Studies not captured by the database search engines were identified through bibliometric cross-referencing. We included studies reporting interventions for respiratory and routinely recommended vaccine-preventable diseases among ethnic minority groups globally. Vaccine-preventable vector-borne, sexually transmitted infectious diseases and non-routinely recommended vaccines were excluded in the search terms (i.e., NOT “HPV or malaria or typhoid or cholera”) (see Supplementary Figure S2), as these vaccines include different considerations to many routinely recommended vaccines. Studies on COVID-19 vaccines were excluded because the original research scope and search were developed before the widespread availability of the COVID-19 vaccine. In addition, the intervention strategies used for COVID-19 vaccines were rapid, and at the time of conducting these searches, the vaccines were still being developed and tested. If a study included groups of diseases in the included and excluded categories, e.g., influenza and COVID-19, only information related to respiratory non-COVID-19 and routinely recommended vaccines were extracted.

### 2.2. Eligibility Criteria

Using PICOS (Population, Intervention, Comparator, Outcome, Study Design), the following inclusion criteria were used:

- Population: studies that included patients and the general public from racial and ethnic minority groups and excluded studies with a majority white ethnic population (i.e., studies with ≥50% white ethnic sample size). Ethnic minority groups were defined as groups that are not part of the majority ethnicity in the country of the study;
- Interventions: reported interventions, which included specific strategies designed to improve vaccination services and uptake in racial and ethnic minority groups, focusing on respiratory and routinely recommended vaccine-preventable diseases. Studies were excluded that did not provide details of the interventions;
- Comparator: included any reported comparator such as pre-intervention data, alternative intervention, or control group;
- Outcomes: studies were included if they reported vaccine behaviour-related data (intention, behaviour and uptake) after implementation of the intervention;
- Study Design: all study designs, including quantitative and qualitative, were included, except case studies and case series.

Only peer-reviewed articles in the English language were included. Papers were excluded if there was no empirical data, if they reported only conference proceedings, or were not in English.

## 3. Screening

Each reference was uploaded to the Rayyan review manager, an app with semi-automation that helped with the initial screening of abstracts and titles based on the eligibility criteria [41]. After the automatic removal of some duplicates, the remaining studies were manually screened. Two investigators (WE and AC) independently conducted initial screening to determine if the eligibility criteria were met. Discrepancies were resolved by discussion between WE, AC and AK. Studies that met the inclusion criteria underwent full-text screening, and the reference lists of all papers included in the synthesis were reviewed for additional articles.

### 3.1. Data Extraction

#### 3.1.1. Intervention Study Details

Data were extracted by WE and AC separately, and 10% of the sample from each individual’s extraction was checked for completeness and quality purposes by another reviewer (AK). For each study, the following data were extracted if available: vaccine focus, study information (including country of study and design); participant characteristics (such as sample size, ethnicity and age); intervention details (such as intervention components and outcomes) and vaccine coverage or uptake, hesitancy, barriers and facilitators.

#### 3.1.2. BCT and BCW Intervention Details

Two reviewers (WE and EG) read the intervention descriptions to identify the BCW components, intervention functions and policy categories reflected within each intervention. The BCTTv1 was used to identify components of the 93 BCTs used in each intervention, which were then categorised into 16 groups. Intervention components were also mapped onto the BCW, nine intervention functions, and seven policy categories. This information (BCW and BCT details) was entered onto a standardised data extraction form. All included studies were coded for the BCTs and BCW by one author (WE) and checked by a second author (EG), and discrepancies were resolved by a third author (AK) to reach a consensus.

### 3.2. Risk of Bias

Risk of bias was measured using the AXIS critical appraisal tool for cross-sectional studies and Critical Appraisals Skills Programme (CASP) guidelines for other study designs [42,43]. The AXIS critical appraisal tool included 20 questions to address study design, reporting quality, and the risk of bias in cross-sectional studies. CASP guidelines for cohort studies, randomised controlled trials, and qualitative research included questions to assess appropriateness of study design, methodology, and results. Each reviewer (WE and AC) assessed 50% of included studies; 10% of these were reviewed by a second reviewer, with discrepancies resolved by a third reviewer (AK). Studies were rated low, moderate, or high. For each study design, the proportion of positive assessments was used to determine the quality of each study. For example, fewer than 11 positive scores using the AXIS critical appraisal tool was considered low quality, 11–16 = moderate, and a score of 17 or higher was high quality (See Supplementary Table S3).

### 3.3. Synthesis Method

Findings from the included studies were entered into tables and descriptively synthesised. The analysis explored the variation in the vaccines reported, study design information, and the intervention details and outcomes. Effect sizes of the outcomes were not accessed due to wide variation in the details reported; this included differences in the measure of effects being used, lack of analysis, heterogeneity of the population samples and insufficient data reporting the same outcome across the studies. Due to the vast differences in the types of interventions, components, measurements, and reported outcomes, a meta-analysis could not be conducted.

In addition, the factors that influenced the intervention implementation and outcomes, such as information on hesitancy, barriers and facilitators to uptake, and challenges experienced while implementing the intervention, were extracted and analysed. Finally, the recommendations suggested by the study authors on approaches to improve similar interventions and other areas that need further exploration were summarised and grouped according to the related BCTs.

## 4. Results

### 4.1. Search Results

**Figure 2:**
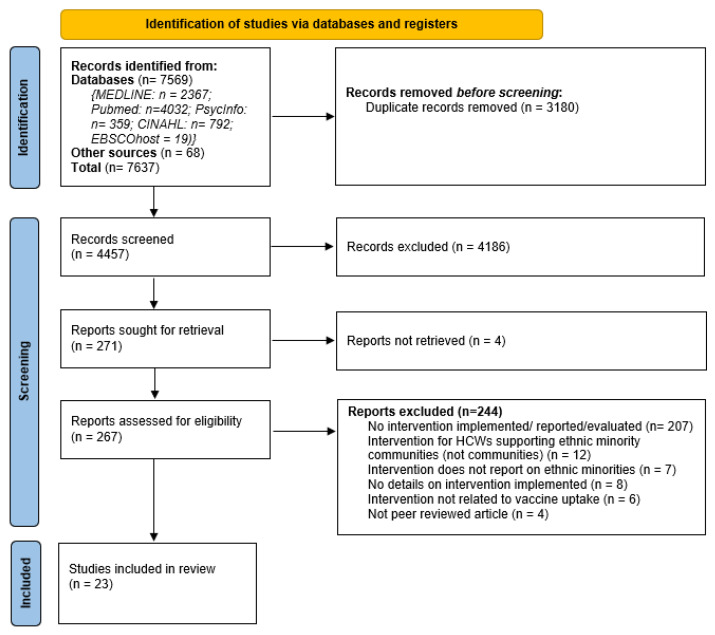
PRISMA flow chart [39].

A total of 7637 citations were identified from searching peer-reviewed databases; 7569 from specific database searches and 68 from hand searching. (Figure 2) Of these, 3180 duplicates were removed, leaving 4457 citations to be screened, of which 271 full texts were reviewed. A total of 35 articles reported vaccination interventions targeting ethnic minority groups, of which 12 studies were on interventions targeted at healthcare workers (HCWs) who support ethnic minority communities. These 12 studies were excluded and analysed as a separate review topic focusing on interventions designed to support HCWs. Another eight studies were later removed due to insufficient information and evidence related to the interventions implemented. Finally, 23 articles reported interventions targeting ethnic minority groups and were included in this review, from which two studies reported the same data [44,45].

### 4.2. Study Characteristics

Table 2 summarises the intervention characteristics and results across the 23 included studies. The selected studies included 166,528 participants from five countries: the United States (*n* =18 studies), the United Kingdom (*n* = 2), and one study from Canada, Greece, and Israel. Ethnic minorities represented were Black/African Americans (*n* = 18), Asians (*n* = 9), Hispanic/Latinos (*n* = 8), two studies on Native American/American Indians and Pacific Islanders, and one study on Jewish and Roma populations (*n* = 1). Study designs used in the included studies were RCT/Quasi-experiments (*n* = 11), cohort (*n* = 6), and cross-sectional (*n* = 6). The vaccines targeted included a wide range of illnesses, including influenza [44,46,47,48,49,50,51,52,53,54,55,56], pneumococcal [47,56], hepatitis [48,57,58,59,60,61,62], polio [48,54,63,64], pertussis [45,48,54,55,56,65], diphtheria and tetanus [45,48,54,55,56], and measles-mumps-rubella [48]. The interventions were conducted in two broad settings: health centres/clinics (*n* = 15) and a community setting (*n* = 8). The interventions were conducted between 2001 and 2021, ranging from two months to five years (min = 5 months, max = 60 months, average = 14.6 months).

### 4.3. Risk of Bias

The studies were of moderate to high quality and had a moderate risk of bias (high = 10; moderate = 13). (Table 2) The potential risk of bias was primarily related to the intervention component details, reporting (e.g., response rates, follow-up, identification of other influencing factors), and statistical methods (including adjustment of confounding factors).

### 4.4. Intervention Effects and Outcomes

As shown in Table 2, outcomes of interest mainly focused on vaccine uptake and coverage amongst ethnic minority patients/participants (*n* = 19 studies). All interventions targeted ethnic minority populations’ behaviour and were designed to access vaccine knowledge and perception [46,55,59,62,63], intention to vaccinate after the intervention [45,49,50,55], adoption of healthy behaviours [62], and cost-effectiveness [54]. Overall, the effects of the different intervention functions and associated BCTs varied. All the studies reported positive changes from the implemented interventions, except the study by Frew et al., 2016 where neither intervention implemented significantly increased influenza immunisation [44].

The study by DiTosto et al., which evaluated whether guidelines were associated with improved Tdap vaccine uptake during pregnancy, reported improvement in vaccine uptake, as women were approximately five times more like to receive Tdap vaccines during the recommended window compared to the pre-guideline cohort (95% CI 3.54–5.72) [56]. A similar intervention aimed to assess women’s intentions to receive influenza and Tdap vaccines during pregnancy and their beliefs about the vaccines and the diseases they prevent after reading evidence-based information about the vaccines. Women were more likely to get the vaccine after reading information: Flu (26.0%), Tdap (49.9%; 43.0% of those in the third trimester) [55]. These findings reflected the positive impact of education, increasing awareness about vaccination, and the personal persuasion felt after receiving and understanding vaccine-related information. Frew et al. described pregnant women’s likelihood of vaccinating their infants against seasonal influenza based on how messages were framed and observed that although a gain-framed message (OR = 2.13, 90% CI = 1.12–4.05) and loss-framed messages (OR = 2.02, 90% CI = 1.08–3.79) were significantly associated with infant influenza vaccination intention compared to control, intention to immunize infants was significantly higher among gain-framed compared to control messages (OR = 2.20, 90% CI = 1.13–4.30), while the loss-framing was not significantly associated with intention to vaccinate infants, compared to gain-framed and control messages [49]. This shows that considering the benefits from vaccination had more influence on increased uptake than what might be lost. In the study to assess the effectiveness of providing personal support to awareness, Wood et al. showed how empowering communities with their personal vaccine information and including follow-up services optimised the immunisation completion rates [54]. In the study, groups with both case management and a health passport had a higher completion rate than those who were only given a health passport (63.8% vs. 50.6%, *p* = 0.01) [54].

### 4.5. Intervention Behaviour Change Components

Behaviour change intervention components targeted Psychological Capability (*n* = 20 studies), Reflective Motivation (*n* = 18), Social Opportunity (*n* = 8), Physical Opportunity (*n* = 7), Physical Capability (n=2), and Automatic Motivation (*n* = 1). (Table 3). Only six of the nine intervention functions were used in the studies; these were Education (*n* = 21), Persuasion (*n* = 17), Enablement (*n* = 9), Environmental Restructuring (*n* = 8), Incentivisation (*n* = 2), and Modelling (*n* = 1). The three intervention functions not identified were Coercion, Training, and Restriction. Five of the six policy categories were also identified; these were Guidelines (*n* = 19), Communication/Marketing (*n* = 17), Service Provision (*n* = 11), Environmental/Social Planning (*n* = 9), Regulation (*n* = 6), and Fiscal (*n* = 2). Legislation was the only policy category not identified in any study.

Twenty-six BCTs were identified across all of the studies, and this represented 12 of the 16 BCTTv1 groups (Table 4). The most commonly used BCTs were Information about health consequences (*n* = 14 studies); “Information about antecedents” and “Credible source” (*n* = 12 each); Prompts/cues (*n* = 9); “Instruction on how to perform the behaviour”, “Restructuring the physical environment”, and “Framing/reframing” (*n* = 7 each); “Social support (practical)” and “Adding objects to the environment” (*n* = 6 each). Twenty studies implemented multiple BCTs ranging from two to nine BCTs [55,59,60], compared to three studies that adopted a single BCT [53,61,64]. On average, across all the studies, approximately five BCTs were used per study. Interventions conducted in community settings (*n* = 8 studies) primarily used an educational approach including coaching [47,48,54,58,59,60,62,66], case management [54], and media-led information and education outreach campaigns [59]. The coaching intervention delivered two approaches: peer coaching with nurse case management (PC-NCM) and peer coaching alone, and compared these with usual care (which included minimal PC and nurse involvement) [60]. The case management intervention also provided health passports to parents, which only contained information on the recommended visits for well-child care and the childhood immunisation schedule [54]. One study after the education intervention provided onsite vaccination [47]; and another study provided community vaccination [66]. Some education interventions used community members to deliver the intervention, which included training peer health educators [48,58] and using religious leaders to enhance recruitment and uptake in vaccination programs [47,58]. Three studies reported providing culturally specific information and interpretation in the local languages of the ethnic minority groups [48,59,66].

### 4.6. Intervention Functions and Policy Categories

The effects of the different identified intervention functions and associated BCTs varied.

#### 4.6.1. Education

Of all the included studies, only two [47,53] did not have the education intervention element. Types of educational resources included brochures and information sheets, video presentations, workshops, community representatives such as church pastors, and public health campaigns. Most of these were aimed at providing information on vaccination and instruction on how to get it. The messages sometimes included the pros and cons of taking vaccination and possible future outcomes.

Armstrong et al. showed that an educational brochure addressing reasons for vaccination refusal was more likely to improve influenza vaccination than only postcard reminders that showed influenza as a leading cause of sickness, hospitalisation, and death in people over 65 years of age, and vaccination timing (66.4% vs. 56.9%, *p* = 0.04). However, video interventions appeared more effective in improving vaccine knowledge than printed media, as shown in three studies [44,45,63]. The video tutorial provided by Frew et al., and Kriss et al. also included information on existing current guidelines for vaccination during pregnancy as a form of providing information about antecedents; this was particularly important, as the most common reason for not taking Tdap vaccines among unvaccinated women was the lack of recommendations from their health physician [44,45].

Interventions expanding to family and close contacts appeared as effective as the individual-targeted strategies common in most studies. For instance, the “Cocooning strategy” to prevent influenza transmission from their close contact with susceptible neonates and young infants in the study by Maltezou et al., provided vaccine services to fathers and other household contacts by first educating them about the safety and efficacy of vaccines and the expected effectiveness for the neonate [51]. From this, higher vaccine uptake during pregnancy was recorded compared to the general US hospital-based post-partum vaccination program (73.7% vs. 44.7% mothers, 55.8% vs. 25.7% fathers, respectively). Similarly, in the UK, Larcher et al. showed that sharing information about health consequences through counselling women positive for hepatitis B (mostly African, Oriental, and Turkish) on the implications for themselves, their partners, and their families, and the need for immunisation of their babies led to a significant increase in baby vaccinations, and a reduction in hepatology referral services during the study [57]. However, another public-focused intervention in the UK using a communication campaign as a means of prompt/cue informed pregnant women about the need for antenatal pertussis and influenza vaccination showed that only 3.0% of the study population (*n* = 6/200) used the public health campaign as the primary source of information, but 16.7% received the vaccine [65]. A similar public campaign in the United States that used a combination of media strategies, including Vietnamese-language versions, emphasising the need for hepatitis B catch-up vaccinations, showed increased awareness of hepatitis B in all study areas, with the significant increase only in the media education area (+21.5% points) vs. control area (+9.0% points) (*p* = 0.001) [59]. These findings evidence direct contact with patients after public strategies, and information in local languages were more likely to increase the effectiveness of a national approach.

Another mass media intervention, a nationwide bOPV campaign in Israel, presented evidence of a large-scale and positive use of prompt/cue [64]. Exposure to media messages was associated with increased vaccine uptake, mostly with 3–5 days delay in all vaccines except for MMRV, which showed a negative effect with 6–8 days delay that diminished afterwards [64]. These observations imply that after increased awareness, vaccine uptake was also influenced by time, as the duration between receiving the information and taking action had an impact on the corresponding actions and outcomes. The time effect illustrated intention to receive vaccination did not always translate to receiving it; for instance, in the intervention by Stringer et al., where nurses provided pros and cons of vaccination through additional information via pamphlets on HBV and offered the immediate vaccine in hospitals when the patients accepted to receive it [61]. Despite the option for immediate vaccine provision, although vaccination acceptance was high (91%), actual vaccination receipt was slightly lower (86%).

#### 4.6.2. Persuasion

The 17 studies that included persuasion intervention functions used a variety of approaches, which included outreach and postcard reminders [46,48], national guidelines [56], communication with healthcare professionals [52,53,60,61,65], support from trained peer health educators and community representatives [48,58,60,67], and visual stimulation through evidence-based videos and reading resources [44,45,50,55]. These approaches reflected all five policy categories identified in this review. Based on methods of delivery, education through video was more persuasive than written sources of information [63].

At a broad scale, implementation of updated guidelines from the Advisory Committee on Immunization Practices (ACIP), which recommended universal Tdap vaccination among pregnant individuals between 27 to 36 weeks gestational age, regardless of prior vaccine status, increased vaccine uptake for Tdap, influenza, and pneumococcal diseases [56]. While at a smaller scale, the studies by Frew et al. illustrated how visual gain-framed messages increased intention to vaccinate compared to loss-framed messages [44,49,50]. This persuasive approach was slightly contradicted in the study by Armstrong et al., which showed providing information about the effect of being unvaccinated as well as addressing related concerns on the dangers of vaccination motivated the participants to seek vaccination [46]. Also, between affective messaging (recommendation to obtain influenza immunisation while acknowledging and discussing patients’ concerns) compared to the cognitive messaging intervention approaches (providing detailed, question-and-answer information on influenza vaccines), no significant increase was observed [44]. Generally, providing some vaccination message, whether gain or loss-focused, encouraged a willingness to get vaccinated compared to no information at all, as illustrated by Frew et al. [49,50]. This highlighted that any form of awareness could increase the intention and receipt of vaccination.

Considering a significant predictor of vaccine acceptance included the history of previous vaccinations, as shown in the study by Schwartz et al. (OR = 8.64, 95% CI = 4.17–17.91, *p* < 0.001), having credible sources like medical assistants and physicians who addressed patients’ concerns and recommended vaccination increased vaccine acceptance (27% agreed to receive influenza vaccination after physicians addressed their concerns) [53]. Nevertheless, healthcare professionals communicating about vaccination did not always significantly increase vaccine uptake [47,65]. For instance, in the study by Donaldson et al., while 47.5% of women indicated a willingness to accept pertussis vaccine in their next pregnancy, 24% had engaged in meaningful discussions with a GP for further information (who were considered credible sources), but 26% received vaccines during the current pregnancy [65]. Observed uptake differed by up to 15.0% between ethnicities, which included Black populations with only 18% uptake [65]. In the study by Callahan et al., 70.2% of Black women reported they would not get vaccinated during pregnancy, and only 23.1% of Black compared to 46.2% of White women unvaccinated in the first and second trimesters planned on receiving a Tdap vaccination [55]. However, engaging community representatives showed to have a positive effect on the intervention, as shown by Daniels et al., where in addition to getting information from credible sources through physician reminders and presentations on vaccination benefits and side effects, church pastors supported the recruitment of people, and this successfully encouraged >90% willingness to participate in education about influenza and pneumococcal vaccination [47]. McPhee et al. also showed that the persuasive approach of using multiple community engagement as practical social support approaches significantly increases the odds of receiving vaccines [community mobilisation (OR: 2.15, 95% CI: 1.16–3.97); media campaign (OR: 3.02, 95% CI: 1.62–5.64)] compared with the control areas for a study on accepting three doses of hepatitis B vaccines [59]. The intervention also provided rewards to children receiving vaccinations. Higher uptake was noted among children who had previously received at least 1 DTP shot, married parents who knew someone with liver disease, and those who had greater knowledge about hepatitis B. Vaccination was, however, still significantly lower for older children.

#### 4.6.3. Environmental Restructuring

Making environmental changes to improve vaccination mainly included using community-based vaccination centres. When onsite vaccinations were offered in a community church, the intervention group received significantly more influenza (OR = 4.8, 95% CI: 2.5–9.4) and pneumococcal (OR = 3.6, 1.8–7.2) vaccinations [47]. In contrast, Donaldson et al. reported low vaccine uptake in healthcare settings (pertussis 34.0% and influenza 48%) [65]. To facilitate attendance immunisations, Larcher et al. added extra components to the existing environment by carrying out weekly vaccine clinics in hospitals at the same time as neonatal follow-up clinics, and also implemented an opportunistic policy for latecomers [57]. From this, 242 infants (91%) were fully vaccinated, and 217 (82%) had serology; hence, full vaccination in the intervention group was 95%, compared to 78% in the control group who were non-residents of the area and often lost to follow-up. The study also reported high mobility among families (25%), significantly affecting outcomes [57]. These findings show that national and clinical interventions adapted to the population were more effective.

To efficiently facilitate the community approaches, tailored resources appeared more successful. Findley et al. used a package of bilingual and community-appropriate immunisation-promotion materials, which supported provider immunisation delivery [48]. The intervention included credible sources, social support, and monitoring elements by using trained peer health educators and personalised immunisation outreach and promotion within social service and educational programs. The intervention resulted in significantly increased immunisation coverage by 11.1% compared to the control, which had none of the intervention services. In addition, 53% of the intervention group were more likely to complete the immunisation series earlier (by 11 days, t = 3.91) compared to the control (AOR = 1.53; 95% CI = 1.33–1.75). The study also stated ethnicity, being Latino, did not significantly influence immunisation coverage (AOR = 1.07; 95% CI = 0.93–1.24). Similarly, the practical social support community mobilisation strategy by McPhee et al. utilised existing health regulation guidelines to establish a Vietnamese-American community-based organisation formed by the coalition that included health workers, local authorities, businesses, and community people and delivered vaccine services [59]. Outcomes from the intervention showed increased receipt of three hepatitis B vaccinations. These findings suggest that community interventions can be effective for any ethnic group. In addition to this, the location of delivery could also influence participation. The community-based participatory research (CBPR) approach used by Ma et al., which utilised churches to inform people about vaccination and offered church health worker training, in combination with more flexible vaccine open clinic hours with bilingual medical staff in the community, recorded a significant increase in HBV screening in the intervention group but not the control [(intervention, 58.5% to 95.8%), control (38% to 39.8%)], with a group difference of 37.8% vs. 1.8% (*p* < 0.001), respectively [58]. Furthermore, there was also an increase in previously non-compliant people’s screening rate in the intervention group (those who never had an HBV test), 93.1%, compared to 2.9% for the control (2 screened/70 never screened) groups. This approach exhibited evidence of the adaptable nature of the environmental/social planning policy category.

#### 4.6.4. Enablement

Strategies used in the nine studies implementing the enablement function included engaging health visitors to meet with families, offering free vaccines, and providing a platform for patients to share their views [47,54,55,56,57,59,60,62,66]. Larcher et al. used hospital-based liaison health visitors as a means of monitoring from credible sources to contact families that did not receive vaccinations and notified their general practitioner, these health visitors attempted to contact the family themselves to reinforce the need for attendance [57]. The community vaccination clinics in the study by Peterson et al. adopted the National Vaccine Advisory Committee standards for immunisation programs in non-traditional settings and used the fiscal measure of obtaining free vaccines through an entitlement program for uninsured children and adults, thereby removing the cost implication from the patients [66]. By using this approach, which provided vaccination instruction and material incentive, through credible sources restructuring physical environments, >80,000 free influenza vaccinations were administered to vulnerable populations over 12 years [66].

The empowerment of patients also served as a means to enable them to make positive vaccination decisions. For example, health passports containing information on the recommended visits for well-child care and approved childhood immunisation schedule were given to all participants in the study by Wood et al. [54]. In addition to the case management assessment of client health, other needs with set goals were included in the intervention package. This approach was used as a means of solving barriers to the receipt of well-child care, such as lapses in insurance or problems with transportation. The results showed immunisation completion in the case management group was 13.2% higher than the control group (63.8% vs. 50.6%, *p* = 0.01), but the high vaccination rate in both groups indicated the benefit of the passport to both groups. Also, Zibrik et al., who conducted 68 socially supportive culturally tailored HBV education workshops over a 12-month period for participants, showed >50% of participants engaged in HBV prevention or management actions, positive behaviour changes to health (62%), and 55% took specific action related to HBV prevention or management [62]. This showed that providing instructions in a community setting on how to take action, in addition to enabling information, had positive effects.

#### 4.6.5. Incentivisation

Only two studies included incentive function in their intervention [58,66]. Ma et al. included the option for community members to train and become health workers, and the study reported increased HBV screening in the intervention group, but not the control [(intervention, 58.5% to 95.8%), control (38% to 39.8%)], with a group difference of 37.8% vs. 1.8% (*p* < 0.001), respectively [58]. As a form of fiscal measure and material incentive, the study negotiated with health providers to lower the cost of HBV tests and treatments for uninsured individuals with HBV infection towards overcoming financial constraints. In the study by Peterson et al., free influenza vaccinations were offered in non-traditional settings for uninsured and underinsured immigrant and ethnic minority groups [66]. Using this approach, 5910 vaccines were administered through 99 community-based vaccination clinics in one influenza season, and of this, 43.1% were uninsured, and 6.9% were vaccinated for the first time. When probed for the reasons for choosing the study clinics, the common reasons given were because it was in a convenient location (19.9%) free vaccination (13.5%), and it provided vaccines for those who lacked health insurance to pay for vaccination (12.8%). This emphasises the relationship between enablement, convenience, and fiscal measures in vaccination interventions.

#### 4.6.6. Modelling

The single study that included a modelling function was by Nyamathi et al., who implemented a three-level peer coaching and nurse-delivered intervention for homeless men recently released on parole from prison and jails [60]. The intervention comprised a series of interactive exercises and role-playing that provided information about vaccination and health consequences, and provided opportunities to practice and rehearse possible scenarios related to vaccination. Each intervention had a research nurse or peer coach who reviewed the vaccine dosing and tracked progress, which was a means of monitoring behaviour without needing direct feedback; however, the nurse involvement varied across the different intervention packages. Peer coaches were former parolees who successfully completed a similar program, and as paraprofessionals, they were positive role models with whom the parolees could identify, therefore, providing social support. All three approaches recorded high rates of HAV and HBV vaccine series completion, 75.4% (PC-NCM), 71.8% (PC), and 71.9% (UC), and the difference in uptake was not statistically significant (*p* = 0.78). This showed that regardless of how involved nurses were, interactive sessions with peer coaches who served as role models helped increase vaccine uptake and completion.

### 4.7. Predictors, Barriers and Facilitators That Influence Vaccination Uptake

Several studies reported factors and predictors contributing to vaccination hesitancy (Table 5). The main predictors of vaccine uptake were history of sickness from past vaccination; knowing someone who became sick; fear of side effects; perceptions around importance and efficacy, such as the inability of vaccines to prevent flu; fear of needles; not wanting it; flu not considered a serious disease; and individuals not wanting to get the flu. In the study by Stringer et al., there were no differences between the acceptors and non-acceptors with respect to their behavioural and attitudinal HBV beliefs [61]. Nyamathi et al. also highlighted a few personal attributes that influenced vaccine uptake, and this included ethnicity (Black and Latinos in the United States), experiencing high levels of hostility, social support, history of injection drug use, ex-prisoners, and people admitted for psychiatric illness [60]. McPhee et al. listed other attributes that decreased vaccination uptake, which included factors such as having older children, higher number of years since the parent immigrated, and household income above the poverty line [59].

#### 4.7.1. Barriers to Vaccine Uptake That Affect Ethnic Minority Communities

The most significant barrier to vaccination was limited knowledge and awareness of vaccination transmission, susceptibility, prevention ability, and the opportunity presented to avoid treatment [45,47,59,62,65]. Other identified barriers included poor accessibility to transportation and inconvenient vaccine appointments, low trust in the healthcare system, lack of culturally relevant and easy-to-understand information, and immigration-related barriers, such as fear of lack of legal status [47,57,62,65]. Also, lack of professional encouragement and uncertainties about the risk and benefits of vaccines acted as barriers to the uptake [45,65].

Behavioural patterns that negatively influence vaccine uptake were based on a perception that a holistic lifestyle, such as a healthy personal lifestyle and breastfeeding, was sufficient for providing needed immunity, so there was a lower risk of contracting the disease [65], and having a previous history of not taking vaccines generally [45,63]. High mobility of families related to address changes significantly affected the effectiveness of hospital-based immunisation programmes [57,63].

#### 4.7.2. Facilitators of Vaccine Uptake in Ethnic Minority Communities

The most significant facilitators identified were awareness of vaccination importance and schedule [54,61] and encouragement or recommendation from healthcare professionals, which was more effective because the HCWs were people they had an existing relationship with [61,65]. Other facilitators included individuals, especially mothers, wanting to take action in the best interests of babies and families by protecting themselves and reducing the risk of a baby developing a disease, knowing someone who had experienced the disease before, having a past personal experience of vaccine-preventable illness, accepting previous vaccines and believing vaccines are effective [50,51,59,65]. The protection of families was a particular motivation for large households [51].

The perception of how serious a vaccine-preventable illness is, and an awareness of how susceptible people are to getting infected, for instance, influenza during pregnancy, also encouraged more vaccination intentions of mothers for their babies [49,50]. Furthermore, normative support from family, friends, healthcare providers, and the community surrounding immunisations increased intention to obtain seasonal influenza vaccine [50,60]. Migration status also influenced uptake, as some migrants had higher vaccine uptake, for example, being of Roma origin or an immigrant [51]. Other positive influencing individual factors included the marital status of parents (married parents had higher vaccination rates), [59] health service accessibility, and affordability [54].

#### 4.7.3. Challenges of Intervention Implementation

Participants with a history of previous vaccination adverse outcomes generally had lower than average vaccination uptake and varied presumptions of the information provided, which was not always in line with recommendations [63,65]. Also, insufficient time to engage in every aspect of the intervention was a challenge, often related to family commitments competing with time for the intervention [45,63]. Hence, some participants relied on HCWs to keep them informed [63]. In addition, Frew et al. [44,50] observed that using a single message exposure was insufficient to persuade or act as a significant factor for determining intention to get immunisations. Less engagement with specific intervention components was also due to low levels of interest; for instance, in the study by Kriss et al., fewer people used the iBook, which was considered less relatable and more challenging to understand than the video [45].

Some operational and financial issues were identified at both hospital and community levels. In hospitals, involving HCWs with different expertise, who are traditionally unfamiliar with the specific vaccinations that were offered, such as neonatologists, paediatricians, nurses, and obstetricians was a challenge [51]. Obtaining health provider reports was also time-consuming and required repeated follow-ups and documentation processes [59]. While at the community level, limited community representative time availability [57] and the qualitative nature of the study did not allow extrapolation of the exact proportion of community members infected with HBV, difficulty in accessing HBV care, or burden from lack of HBV knowledge [62]. In the study by McPhee et al., it was not possible to determine the eligibility of some participants who were unreachable or refused to be interviewed [59]. Loss for follow-up due to difficulty in contacting non-attenders with name changes also affected the Larcher et al. study [57]. This was similar to the limited information available to HCWs to allow accurate identification of patients, and very few could find records reporting vaccination dates [59]. Stringer et al. also noted that there was no data on the completion of the entire vaccine series; therefore, the study population cohort may not be substantively protected. However, acceptance rates were likely to be higher when the vaccination was offered as part of the usual care, for instance, pregnant adolescents [61]. Also, there was a need to offer several vaccination opportunities, including during evening hours or weekends [51].

A few intervention components were stopped before the end of the study. For instance, the insurance pilot program where insurance payment would be accepted only for people with vaccination insurance showed that accounting outweighed the benefits, and was discontinued in the study by Peterson et al. [66]. Also, Schwartz et al. reported that vaccine supplies were exhausted before the expected study end date [53]. Financial constraints to sustain the interventions were challenging [51,54,57]. For example, Wood et al. reported that the overall cost-effectiveness of the intervention (case managers for infants < 6 weeks and home visits two weeks prior to scheduled immunisations) compared unfavourably with other medical interventions [54]. Also, Nyamathi et al. were concerned that the policies enacted in the California state prison system, particularly realignment (or reducing the state prison population by transferring inmates to county jails), would affect vaccination completion [60].

## 5. Discussion

This systematic review identified 23 studies across five countries that reported interventions aimed at increasing vaccination uptake among ethnic minority populations. All the studies reported that the interventions were somewhat effective, with varying improvements in vaccine uptake and/or reduction in hesitancy. Twenty-six BCTs, six BCW intervention functions, and seven policy categories were identified to promote vaccine delivery and uptake. The most common BCTs used in at least six studies were Information about health consequences (to increase risk awareness and vaccine knowledge), Information about antecedents (to advise patients about vaccination impacts), Credible source (to provide guidelines and health workers’ collaboration with community organisations), Prompts/cues (to provide reminders and motivation), Instruction on how to perform the behaviour (to present guidance on how to get vaccinated), Restructuring the physical environment (to provide ease of access to vaccine service), and Framing/reframing (to present information in formats that can motivate change).

Some intervention functions appeared more effective in improving vaccination rates; however, the most effective were those with a community-based component, which included community clinics and the offer of free vaccination. Predictors of vaccine uptake often acted as both barriers and facilitators. The main predictors included the experience of sickness after previous vaccination and fear of side effects, which were related to barriers and linked to limited knowledge of vaccination transmission, low perceived susceptibility, and low vaccine efficacy. Behavioural patterns linked to positive attributes sometimes negatively influenced vaccine uptake. For instance, a holistic lifestyle (such as healthy eating and breastfeeding) was perceived to be sufficient for providing immunity against vaccine-preventable diseases. These barriers were largely overcome through interaction, encouragement or recommendations from healthcare professionals, which was a key facilitator for increased vaccination uptake. Hence, despite intervention challenges, such as the high resource cost (financial cost and vaccine supply) needed to deliver some interventions, recommendations for future interventions in these studies included the need for additional resources to enhance social support, in addition to providing vaccine education that was locally and culturally relevant to the target community [51,54,62].

Previous studies have reported psychological predictors of vaccination, including vaccination history, perception of disease severity, and vaccine safety and effectiveness [4,9,68,69]. To address these challenges, as identified from this review, positive changes in beliefs about vaccinations can be achieved through increased awareness by targeting individuals or groups directly, compared to public campaigns, and where public campaigns are used, it is important to follow-up with direct contact with individuals and groups in a timely manner [70]. The most effective way of sharing information was visual modes of delivery, which were more effective than written communication; sharing information in this way enabled participants to identify with the characters in the resource [45]. Also, although most messages can facilitate increased vaccine uptake, positively framed messages have more influence in increasing vaccination [49,50]. Previous studies have shown that negatively framed messages aimed to elicit feelings of anticipated regret for not getting vaccinated were generally perceived as patronising and unprofessional [71]. Hence, it is important that messages are factual and emphasise the costs and benefits of vaccination, thereby showing that two-sided messages are viewed more credibly [72]. Furthermore, having messages from credible sources like physicians did not always significantly increase vaccine awareness and intention to receive a vaccine, but in combination with a trusted community representative, such as a church pastor, more community members can be motivated to consider taking vaccines [70].

Although mass media campaigns can reach a broad spectrum of any population, their effects remain modest, primarily because of the effect gaps between intention-to-action [73,74]. This can be addressed through the use of personalised reminders and prompts [75]. Furthermore, mass vaccine clinic venues often do not suit some ethnic, cultural or faith-based minority groups, especially where privacy and time are required for meaningful dialogue [13]. Poor understanding of the health system, language, cultural barriers, and poor doctor-patient relationships compound access issues in minority groups [76]. Some barriers may stem from longstanding structural inequities and the fact that some racial and ethnic minority communities live in areas of higher deprivation with large family sizes, low-income levels, and a higher burden of diseases, which was identified as a risk factor during the COVID-19 pandemic [76,77,78]. Adapting existing services and guidelines to match community preferences, for instance, offering free services through community-based clinics compared to hospitals to deliver vaccines, emphasises the interrelationship between different intervention functions (incentivisation, environmental restructuring, enablement and fiscal measures) and how using multi-intervention functions for vaccination services can improve the effectiveness of services provided.

### 5.1. Limitations of the Review

This review may be subject to publication and selection bias, as unsuccessful interventions may be less likely to be documented in peer-reviewed literature. The review is also limited in its focus on only respiratory and routinely recommended vaccines, which means the findings should be interpreted with caution for non-respiratory and routinely recommended vaccines. Study inclusion is likely to have been restricted due to use of PICO questions, which emphasise specific, single-component strategies, whereas many intervention strategies are neither designed nor evaluated in this way. Multi-component interventions were identified, but only overall impact data were presented. Therefore, outcome data for individual strategies to address vaccine hesitancy were not separately available.

There is evidence of vaccine hesitancy in all populations in different countries, but this is greater evidence among some ethnic minority populations [4,13]. Yet, only five countries were represented in this review, with most studies from the United States. No relevant study reporting interventions to support ethnic minority groups in Asia and Africa was identified. This indicates an evidence gap, on a global scale, about vaccinations for racial and ethnic minority groups. More research which includes racial and ethnic minority populations is required to ensure evidence-based vaccine interventions are developed that reflect the needs of all communities, including diverse groups. In addition, studies on COVID-19 vaccines were excluded as the aim of the study was designed, in part, to inform strategies to support the existing and future COVID-19 vaccination programme. However, inclusion of several non-COVID-19 respiratory vaccination interventions includes sufficient parallels and considerations for the COVID-19 vaccination programme.

### 5.2. Recommendations

The present review findings suggest that increased awareness and knowledge sharing from credible sources have the potential to encourage the general public to get vaccinated, but this needs to be community focused. It is also essential to strengthen engagement and build trust with ethnic minority communities, and acquire a better understanding of how to support diverse groups by ensuring more meaningful inclusion through more culturally competent health systems. This approach, especially for addressing COVID-19 hesitancy, would require co-producing solutions based on the principles of inclusion and engagement. This can be guided using three core elements as suggested by Chevallier et al., which are by testing a communication campaign addressing vaccine hesitancy, using behavioural insights to make vaccination more accessible, and leveraging the power of social norms [73]. To effectively design a community-based intervention, members of the community also need to be included. For instance, Frew et al. showed that engaging with potential participants in the formative stage of research provided additional insights into the content of the framed message, and this contributed to a high level of engagement in the final intervention [49]. Table 6 provides an overview of recommendations to develop vaccine interventions for racial and ethnic minority groups based on the findings of this review. The use of behavioural science frameworks such as the COM-B model, BCW, and BCT can guide the development of interventions which are tailored to the motivational drivers, educational needs, and which reflect the socio-cultural context of diverse communities. Table 6 outlines recommendations based on BCTs identified in this review that can be used to promote vaccine uptake in racial and ethnic minority communities.

## 6. Conclusions

The relevance and effectiveness of vaccination strategies are critical for successful public health protection against infectious diseases. To optimise outcomes, all members of the population need to be engaged, and this includes racial and ethnic minority populations. Identifying the intervention components and behaviours that make effective and efficient services are essential for proper planning and implementation. This systematic review has shown which vaccine strategies work well and the factors that encourage vaccine uptake. The most common approach used is related to education and providing vaccine information to targeted populations, and this is most effective when provided in a visual format, delivered through credible sources which include healthcare professionals and respected community representatives, repeated exposure and providing follow-up opportunities for dialogue in a timely manner. To design effective interventions related to the approach identified in this review, strong support from government and healthcare organisations would be needed to institute tailored, culturally appropriate approaches, as there is no one-size-fits-all solution and vaccine strategies have to be adapted according to the different needs of the ethnic minority population.

## Figures and Tables

**Table 1:** Search terms used to identify relevant publications for the review.

Concept	Key Words
Vaccine hesitancy	vaccin* AND (hesitan* OR refus* OR confiden* OR accept* OR uptake* OR adopt*)
Minority ethnic groups	(ethnic group*) OR (ethnic minorit*) OR (minority group*) OR ethnic* OR minorit* OR race* OR racial OR Black* OR African* OR Asian* OR (South Asian*) OR Bangladeshi* OR(Pakistani*) OR Japanese OR Chinese or Korean* OR Arab* OR BME OR BAME OR Roma* OR Hispanic* OR Caribbean* OR (people of color) OR (person of color) OR Jewish OR Jews OR gyps*
Intervention	Interven* OR communicat* OR train* OR motiv* OR strateg* OR guid* OR program* OR support* OR polic* OR approach* OR procedure* OR plan* OR engag*

**Table 2:** Description of vaccine interventions implemented in increased vaccine uptake among EM communities.

Study	Vaccine Focus	Study Design/ Study Period/ (Intervention Duration)	Country(s) of Study	Reported Ethnic Minority Group	Setting/Study Population Sample	Intervention/ Comparison (Control)	Intervention Outcomes	Vaccine Coverage/Uptake	Study Quality
**Armstrong** **et al., 1999 [46]**	Influenza	RCT 1996–1997 (7 months)	United States	African American	Primary care clinic *n* = 740 community-dwelling individuals	Education brochure Mailed postcard reminder **Comparison** Mailed postcard reminder without educational content	- Educational brochure (229 individuals) vs. Postcard reminder (202 individuals). - Educational brochure group was more interested in influenza vaccination in coming year (66.5% vs. 57.1%, *p* = 0.05).	Educational brochure group more likely to be vaccinated than postcard reminder group (66.4% vs. 56.9%, *p* = 0.04)	Moderate
**Callahan** **et al., 2022** **[55]**	Influenza, Tetanus, Diphtheria, Pertussis (Tdap)	Cross-sectional (Survey) 2019–2021 (15 months)	United States	Black	Hospital *n* = 664 women	Reading informational text	**Influenza vaccination during pregnancy:** 52.5% already vaccinated, 10% planned to be vaccinated, 3.1% planned to be vaccinated after giving birth; 34.0% did not plan to receive the influenza vaccine this season, 0.4% did not answer **Tdap vaccination during pregnancy:** 25.5% already received; at 3rd trimester (63.7% vaccinated; 30.9% not planning to be vaccinated), 37.8% of unvaccinated planned to be vaccinated **Ethnicity** - **Flu vaccine:** 70.2% Black women would not get vaccine during pregnancy - **Tdap vaccine:** Unvaccinated in 1st and 2nd trimester planning to be vaccinated (Black 23.1%, White 46.2%) **Communication** - **Flu unvaccinated:** 19.8% would ask HCP; 9.6% interested in educational materials - **Tdap unvaccinated:** 26.9% interested in educational materials - **Found information text useful:** 55.5% of flu unvaccinated, 66.0% Tdap unvaccinated - **Likely to get vaccine after reading information:** Flu (26.0%), Tdap (49.9%; 43.0% of those in 3rd trimester)	NR	High
**Daniels** **et al., 2007** **[47]**	Influenza, Pneumococcal	RCT 2003–2004 (2 months) 2005–2006 (4 months)	United States	African American Asian Latino	Community (Faith based-Churches) *n* = 330	Vaccine educationOn-site vaccination **Comparison** Vaccine education only	- Intervention group more likely to receive influenza vaccinations (OR = 4.8, 95% CI: 2.5–9.4) and pneumococcal vaccination (OR = 3.6, 1.8–7.2) - >90% reported willingness to participate in education and promotion programs	**Vaccine utilisation** - **Influenza:** Intervention (80%, 90/112), control (46%, 32/70), *p* = 0.001 - **Pneumococcal**: Intervention (66%, 58/88) control (35%, 20/56), *p* = <0.001	Moderate
**DiTosto** **et al., 2021** **[56]**	Influenza, Tetanus, Diphtheria, Pertussis (Tdap) Pneumococcal	Cohort (Retrospective) 2011–2015 (4 years)	United States	Non-Hispanic White Non-Hispanic Black Asian Hispanic	Hospital *n* = 2294 women	Guideline	**Received prenatal care post-guideline:** 70.2% (*n* = 1610/2294) **Post-guideline cohort:** - Less likely to have initiated prenatal care during 1st trimester (81.2% vs. 90.1%, *p* < 0.001) - Fewer prenatal visits compared to pre-guideline cohort (11.2 ± 4.7 vs. 13.3 ± 4.0, *p* < 0.001) - Higher frequency of receiving Tdap vaccine during pregnancy or postpartum compared to pre-guideline cohort (*n* = 1385, 86.1% vs. *n* = 322, 47.4%, *p* < 0.001) - Care associated with 7.37-times greater odds of receiving Tdap vaccine during pregnancy or postpartum compared to care pre-guideline cohort (95% CI 5.93–9.18). - Receiving Tdap vaccine between recommended time (27–36 weeks gestational age) improved from 52.5% to 91.8% after guidelines (*p* < 0.001). - 4.50-times greater odds of receiving Tdap vaccine during recommended window compared to pre-guideline cohort (95% CI 3.54–5.72) - Receiving influenza vaccine during pregnancy more frequent than pre-guideline cohort (*n* = 1159, 72.0% vs. *n* = 419, 61.2%, *p* < 0.0001). - No significant difference in uptake of the pneumococcal vaccine (*p* = 0.12; AOR 2.00, 95% CI 0.93–4.79) - Receiving Tdap vaccine during the recommended gestational age window associated with race and ethnicity (*p* = 0.017), null parity (52.8% vs. 43.8%, *p* = 0.036) and increased number of prenatal visits (11.3 ± 4.7 vs. 9.5 ± 4.7, *p* < 0.001). - Receiving influenza vaccine during pregnancy significantly associated with older age (33.4 ± 4.7 years vs. 32.8 ± 5.1 years, *p* = 0.017), race and ethnicity (*p* = 0.017), initiating prenatal care in the first trimester (76.9% vs. 66.8%, *p* < 0.001), and increased number of prenatal visits (11.4 ± 4.6 vs. 10.7 ± 5.2, *p* < 0.001)	**Vaccine uptake pre- and post-guideline** - **Tdap:** 47.4% to 86.1%, *p* < 0.001 - **Influenza: **61.2% to 72.0%, *p* < 0.0001 - **Pneumococcal: **3.8% to 7.3%, *p* = 0.12	High
**Donaldson** **et al., 2015** **[65]**	Pertussis	Cross-sectional 2013–2014 (14 months)	United Kingdom	Asian Black	Healthcare centre/clinic *n* = 200 women	Communication campaign **Comparison** Standard routine vaccination	- **Awareness of programme**: 63% (126/200) - **Willingness to accept pertussis vaccine in next pregnancy (*n* = 200)**: yes (47.5%), undecided (38.5%), do not wish to take up the vaccine (8.0%), no answer (6.0%) - Information received from multiple sources, primarily General Practitioners (GP) and midwives, but included relatives and friends, media sources (printed material, radio, and the internet) - 3.0% (6/200) used public health campaign as primary source of information; only one (16.67%, 1/6) received vaccine - **Vaccine offered at GP practice**: pertussis (34.0%, 68/200), influenza (48%, 96/200) - Among those who met with GP for pertussis vaccine or further information: 24% (48/200) engaged in meaning discussion, 61.5% not offered, 4.5% could not remember - **Informed of vaccination**: pertussis (38.8%, 49/126 declined); influenza (50.3%, 70/139 accepted during pregnancy) - Uptake differed by up to 15.0% between ethnicities. - **Complications in current pregnancy **21.5% (43/200): [gestational diabetes (13, 6.5%); pre-eclampsia (7, 3.5%)] - **Not been vaccinated (*n* = 144):** 79 (54.8%) undecided about accepting the vaccine during this pregnancy, but may consider it in the future	- **Actual vaccine uptake during current pregnancy**: 26.0% (52/200) - **Ethnic differences**: Highest uptake “White women” (29.5%, 26/88), mostly “White–Other” pre-dominantly Polish, (36.0%, 18/50); Lowest uptake “Black/Black British” (18.9%, 7/37), lowest in Black Caribbean (7.1%, 1/14); No reported ethnicity (14.3%, 1/7) - **Uptake by pregnancy complication**: complicated pregnancy (30.2%, 13/43) vs. uncomplicated pregnancies (22.9%, 36/157)	High
**Dunn** **et al., 1998** **[63]**	Polio	RCT 1997 (4 months)	United States	African-American Hispanic/Latino Asian Native American	Paediatric offices and local health department immunization clinic *n* = 287 parents/guardians	Education **Comparison** Usual routines with respect to presentation of VIS and vaccine discussions and recommendations with the parent/guardian	- Both interventions increased knowledge test scores. - Videotape viewers scored higher compared to VIS only: across all health practice types (*p* < 0.05), racial/ethnic groups (African-America, *p* < 0.001; Hispanics, *p* = 0.07), and educational levels (increased with increasing educational level, *p* < 0.001) - **Read VIS**: Videotape + VIS group (41%, 58/143), VIS-only (62%, 89/144). - **Videotape + VIS group**: Reading VIS did not improve scores for videotape viewers; videotape more helpful than VIS (43%, 25/58); videotape and VIS equally helpful (53%, 31/58); did not read VIS but preferred videotape (8%, 11/143). - **VIS group**: Reading VIS improved scores of VIS only group, but not as high as for videotape viewers who did not read the VIS; VIS effective in providing information (98%, 83/89)	NR	Moderate
**Findley** **et al., 2008** **[48]**	Diphtheria, Tetanus, Pertussis, Polio, Measles, Mumps, Rubella, Haemophilus Influenza, Hepatitis	Cohort (Retrospective) 2006–2007	United States	Latino	Community *n* = 10,857 children	Outreach Education Reminders **Comparison** No intervention	- Intervention group completed immunization series earlier, by 11 days (t = 3.91); 53% more likely to be up-to-date than control (AOR = 1.53; 95% CI = 1.33–1.75). - Neither Latino ethnicity (AOR = 1.07; 95% CI = 0.93–1.24) nor Medicaid (AOR = 1.05; 95% CI = 0.95–1.16) significantly influenced immunization coverage.	- Intervention higher immunization coverage (11.1%) than control children (χ^2^ = 44.6, *p* < 0.001)	High
**Frew** **et al., 2013** **[49]**	Influenza	RCT 2011–2012 (9 months)	United States	Black/African American Hispanic/Latino	Clinics *n* = 261 pregnant women	Message framing **Control** Usual care	- **Formative research recommendation**: Gain-framed message (four lines of factual information about influenza vaccination with visual background of smiling pregnant woman); Loss-framed message (four lines of text emphasizing risks of not protecting oneself and unborn child(ren) from influenza with background of an ambulance and stretcher). - **Intervention (*n* = 261)**: Gain (*n* = 87), Loss (*n* = 90), Control (*n* = 84). - 50.2% (131/261) indicated intention to vaccinate their new infant after 6 months of age. - Gain (OR = 2.13, 90% CI = 1.12–4.05) and loss-framed messages (OR = 2.02, 90% CI = 1.08–3.79) significantly associated with infant influenza vaccination intention compared to control. - Intention to immunize infants significantly higher among gain-framed compared to control messages (OR = 2.20, 90% CI = 1.13–4.30). - Loss-framing not significantly associated with intention to vaccinate infants, compared to gain-framed and control message. - Intention to immunize during pregnancy had a strong effect on intent to immunize infants (OR = 10.83, 90% CI = 4.92–23.83). - **Viewed feature film “contagion” (20.69%, 54/261)**: viewed gain- and loss-framed messages as appealing (χ^2^ = 6.03, *p* = 0.05), novel (χ^2^ = 16.33, *p* = 0.03), easy to remember (χ^2^ = 6.24, *p* = 0.0003). - Few in gain- and loss-arms tired of the type of messages presented (χ^2^ = 9.31, *p* = 0.01) or the message “left them cold” (χ^2^ = 6.23, *p* = 0.04) compared with those who did not see the film but were exposed to the very same messages. - Ethnicity not a significant contributing factor in maternal intent to immunize infants	NR	Moderate
**Frew** **et al., 2014** **[50]**	Influenza	RCT 2011–2012 (9 months)	United States	Black/African American Hispanic/Latino	Clinics *n* = 251 women	Message framing **Comparison** Control messages	- **Likelihood of obtaining influenza immunization during pregnancy**: Gain-framed group (OR = 1.19, 90% CI = 0.45–3.14), Loss-framed group (OR = 0.58, 90% CI = 0.22–1.55)	NR	Moderate
**Frew** **et al., 2016** **[44]**	Influenza	RCT 2013 (4 months)	United States	Black/African American	Antenatal clinic *n* = 65 pregnant women	Video **Comparison** 34 participants-comparison condition (receipt of the Influenza Vaccine Information Statement)	- **Baseline**: 63% (60/95) not received seasonal influenza immunization during the previous 5 years; low likelihood immunization in current pregnancy (2.1 + −2.8 on 0–10 scale). - Neither intervention format (Arm 2 or Arm 3) significantly increased influenza immunization. - **30-days postpartum follow-up**: no effect observed after single exposure to either “Pregnant Pause movie” affective messaging (RR = 1.10; 95% CI = 0.30–4.01) or “Vaccines for a Healthy Pregnancy iBook” cognitive messaging (RR = 0.57; 95% CI = 0.11–2.88). - **Reasons for not obtaining maternal influenza immunizations (*n* = 85):** Concern about vaccine harm (47%), low perceived influenza infection risk (31%), history of immunization non-receipt (24%), vaccine was not recommended to by doctor (18%), don’t think the vaccine works or works well (15%)	Arm 1 (comparison group) = 12% (4/34) Arm 2 (pregnant pause movie) = 13% (4/31) Arm 3 (vaccines for a healthy pregnancy iBook) = 7% (2/30)	Moderate
**Kriss** **et al., 2017** **[45]**	Diphtheria, Tetanus, Pertussis	RCT 2013 (4 months)	United States	African American	Antenatal clinics *n* = 106 women	Messaging video and a cognitive messaging iBook **Comparison** Standard CDC Vaccine Information Statements (VIS)	- **Perinatal Tdap vaccination**: Control (18%), iBook group (50%), (RR [vs. control] = 2.83; 95% CI = 1.26–6.37); video group (29%) (RR = 1.65; 95% CI = 0.66–4.09) - Intention to receive Tdap in next pregnancy improved in all three groups	Prenatal Tdap vaccination: Control (18%), iBook group (50%), video group (29%)	High
**Larcher** **et al., 2001** **[57]**	Hepatitis B	Cross-sectional (Retrospective) Babies born 1992–1996 (5 years)	United Kingdom	Black African Caribbean Asian Turkish Vietnamese	Hospitals (clinic) *n* = 265 infants born to hepatitis B carrier mothers	National program (Hackney residents) **Comparison** Hackney non-residents	- 242 infants (91%) fully vaccinated; 217 (82%) had serology; 31 required booster doses. - Failing to reach 2nd, 3rd vaccinations and serology on schedule rose exponentially (7%, 18%, 33%, respectively). - **Received a full vaccination in non-hospital based primary care**: Tower Hamlets (7 of 22 babies), Hackney (53 of 58 babies). - **Time of 1st vaccine administration (*n* = 184 infants):** within 48 h of birth (180, 98%), within 24 h (164, 89%). - **Requirements for specific postnatal counsellin**g of mothers and hepatology referral fell significantly during the study. - **Translation requirements**: High (85% for Turkish, Vietnamese, and Asian families). - **Mobility**: high (25%) and significantly affected outcome.	- **Fully vaccinated: **91% infants. - **Fully vaccinated babies**: Hackney residents (95%), non-residents (78%).	Moderate
**Ma** **et al., 2012** **[58]**	Hepatitis B	Mixed methods (Quasi-experiment (RCT), interview, survey, workshops) 12 months	United States	Korean American (Asian)	Community (Faith based-Churches) *n* = 330	Community-based participatory research (CBPR) **Comparison** Concurrent control group with no intervention offered	Short-term intervention effects of primary outcomes - **Screening conversion rate:** 90.2% (93.1% vs. 2.9%). - Increased HBV screening in intervention group not control: (intervention, 58.5% to 95.8%), control (38% to 39.8%); group difference, 37.8% vs. 1.8% (*p* < 0.001), respectively.	**Vaccination prevalence rate:** 33% (33% vs. 0%)	High
**Maltezou** **et al., 2012** **[51]**	Influenza	Cohort (Prospective) 2011–2012	Greece	RomaImmigrants	Tertiary hospital (clinic) *n* = 224 mothers	Household vaccination recommendation	- **Received the influenza vaccine (*n* = 242 neonates):** mothers (73.7%, 165/224), fathers (55.8%,125/224), higher than uptake during pregnancy recorded in a US hospital-based post-partum vaccination program (mothers, 44.7%; fathers, 25.7%). - **Significant factors associated with increased vaccination rates among mothers:** being of Roma origin (*p* = 0.002), being an immigrant (*p* = 0.025), giving birth to a neonate with birth weight <2500 g (*p* = 0.012), and residing in a family with ≥4 family members (*p* = 0.017). - Neonates’ father with vaccinated mothers 6-fold higher vaccination rates compared those with mothers who refused vaccination (*p* < 0.001).	- Influenza vaccine administration: 46.9% (348/742) household contacts of 242 neonates (including mothers (73.7%, 165/224), fathers (55.8%, 125/224).	High
**McPhee** **et al., 2003** **[59]**	Hepatitis B	RCT 1998–2000 (2 years)	United States	Vietnamese American (Asian)	Community *n* = 2648 1547 parents; 1101 providers (for Children 3 to 18 years)	Media-led information and education outreach campaigns. **Comparison** Children living in the control (Washington, DC) area. different location so received none of the interventions.	- **Awareness of hepatitis B**: increased between the pre- and post-intervention surveys in all three areas, significant increase only between media education area (+21.5% points) and control area (+9.0% points) (*p* = 0.001). - **Post-intervention**: More parents knew free vaccines were available for children in media education (+31.9%) and community mobilization (+16.7%) areas than control area (+4.7%), both (*p* = 0.001). - **Knowledge of sexual transmission of hepatitis B virus**: Higher increase in the media education area (+14.0% points) and community mobilization (+13.6% points) areas compared with control area (+5.2% points). - **Odds of receiving three hepatitis B vaccine doses:** significantly greater for both community mobilization (OR: 2.15, 95% CI: 1.16–3.97) and media campaign (OR: 3.02, 95% CI: 1.62–5.64) interventions compared with the control area. - **Odds of vaccination**: significantly greater for children who had had at least 1 DTP shot, married parents who knew someone with liver disease, heard of hepatitis B, and had greater knowledge about hepatitis B. Vaccination odds significantly lower for older children.	**Receipt of 3 hepatitis B vaccinations**: increased in community mobilization (26.6% to 38.8%) and media intervention (28.5% to 39.4%) areas; declined in control community (37.8% to 33.5%)	Moderate
**Nicoleau** **et al., 2001** **[51]**	Influenza	Cohort (Prospective) 1999 (2 months)	United States	African American	Private clinic *n* = 231	Discussion with physician **Comparison** Before discussion with physician	- 33% initial vaccine decliners changed their minds (41/123) after discussion with physician. - 44% (98/221) intended to be vaccinated before discussion with physician, and rose to 63% (139/221) after discussions with HCP about potential side effects, efficacy, and safety. - Uptake compares favourably with national health objective of 60% or greater for year 2000.	NR	High
**Nyamathi** **et al., 2015** **[60]**	Hepatitis A and B	RCT 2010–2013 (12 months)	United States	African American Latino Asian/Pacific Islander	Community *n* = 600 recently paroled men	Coaching **Comparison** None (or “Usual care”)	- 345 participants eligible for HAV/HBV vaccine, predominantly African–American (51%) and Latino (31%) - **Vaccine completion rate for interventions**: 75.4% (PC-NCM), 71.8% (PC), and 71.9% (UC) (*p* = 0.78)	**Vaccine completion rate for ≥3 doses**: 73% among all three interventions	Moderate
**Peterson** **et al., 2019** **[66]**	General	Cross Sectional (Program report) 2006–2017 (3 years)	United States	Black/African Asian/Pacific Islander Hispanic/Latino American Indian	Community (Faith based-Churches) *n* = 5910	Community-based vaccination clinics	- **Year 2006 to 2018:** provided >80,000 free influenza vaccinations to vulnerable populations. - **1st time vaccinated for influenza **(*n* = 5910): Yes (6.9%), No (91.3%), Not specified (1.8%). - **Reasons for choosing MINI clinic **(up to three reasons per respondent, *n* = 8561 responses): convenient location 19.9%, free vaccination (13.5%), lack of health insurance to pay for vaccination (12.8%) - **Source of information about clinic **(*n* = 5910 respondents): faith community (49.0%), friend or family member (13.1%), community agency/site (9.6%), fliers (5.4%)	5910 first time vaccine administered through 99 community-based vaccination clinics (uninsured (43.1%))	Moderate
**Sagy** **et al., 2018** **[64]**	Polio	Cross-sectional (Retrospective) 2013 (3 months)	Israel	Jewish Non-Jewish	Medical records (clinic) *n* = 138,799 OPV vaccines administered	National programme	- **Vaccines administered**: DTaP-HIb-IPV (1280), MMRV (1018), PCV (660), RVV (636). - **Media exposure:** associated with increased vaccine utilization, mostly with 3–5 days delay in all vaccines except MMRV (negative effect with 6–8 days delay, which afterward diminished). - **Most prominent associations**: among Jews and high SES groups (RR = 1.33, 95% CI = 1.06–1.67), DTaP- HIb-IPV vaccine (RR 1.36; 95% CI = 1.08–1.71), RVV vaccine for Jews (RR = 1.27, 95% CI = 1.01–1.60) for high SES within PCV vaccine. - Positive media exposure associated with increased bOPV uptake, mostly in 3–5 day lags. - Negative media exposure not associated with change in vaccines uptake. - 80–90% coverage reached in District where outbreak begun among the high-risk paediatric population.	138,799 bOPV vaccines given (80–90% coverage)	Moderate
**Schwartz** **et al., 2006** **[53]**	Influenza	Cross-sectional (Prospective) 2003–2004 (4 months)	United States	African American	Primary care clinics *n* = 454 patients	Medical assistant (MA)-initiated universal standardized vaccination	- Similar proportions of African Americans and white groups received 2003 vaccine (11.6% and 11.0%, respectively), had vaccination as the reason for visit (23.8% and 30.5%, respectively) - **Accepted vaccination during intervention**: African-American (62.1%), white (68.9%). - **Significant predictor of vaccine acceptance:** History of previous vaccination (OR = 8.64, 95% CI = 4.17–17.91, *p* < 0.001); but not significant for white and African American groups after adjusting for confounders (AOR = 1.20, 95% CI = 0.63–2.29, *p* = 0.57). - Physician addressed concerns about vaccination (*n* = 59 patients); 27% (16/59) agreed to receive influenza vaccination.	African–American (62.1%) White (68.9%)	High
**Stringer** **et al., 2006** **[61]**	Hepatitis B	Cohort 1999–2000 (12 months)	United States	African American	Tertiary clinic *n* = 160	Information pamphlet Reoffering vaccine	- Acceptance of vaccination (91%, 146/160). - Reoffering vaccine a successful intervention, even with adolescents with less-than-optimal prenatal attendance.	Actual vaccination uptake (86%, 131/154)	Moderate
**Wood** **et al., 1998** **[54]**	Diphtheria Tetanus Pertussis vaccinations, Polio Haemophilus Influenza B	RCT 15 months	United States	African American	Community *n* = 419 infants	Case management Health passport **Comparison** Health passport only	- **Immunization completion:** case management group higher than the control group (63.8% vs. 50.6%, *p* = 0.01). - Case management effect limited to 25% of sample reporting three or fewer well-child visits (OR = 3.43, 95% CI = 1.26–9.35), and immunization increased by 28%.	**Immunization completion:** - Case management group (63.8%). - Control (50.6%)	Moderate
**Zibrik** **et al., 2018** **[62]**	Hepatitis B	Cohort (Mixed methods) 2014 (12 months)	Canada	Asian/South Asian	Community *n* = 827	Workshop	- >50% of HBV education workshop participants engaged in HBV prevention or management actions. - Awareness campaign reached >11,800 individuals recorded through public education workshops attendance and receipt of educational materials. - Self-reported changes to lifestyle and HBV prevention/management from the 2-week and 1-month follow-up interviews. - **Follow-up interviews (*n* = 633)**: Positive behaviour change to health (62%, 391/633), Taken specific action related to HBV prevention or management (55%). - **Checked vaccination status** for self or family member: 6% (22/352); got vaccinated against HBV: (1%, 4/352).	**Vaccinated against HBV:** 41.3% (*n* = 331)	High

Note: AOR = Adjusted Odds Ratio; GP = General Practitioners; HBV= Hepatitis B virus, HCP = Health Care Professional.

**Table 3:** Summary of the BCW components identified in the vaccination interventions.

Study (Author, Year)	Components			Model of Behaviour						Intervention Function									Policy Categories						
	Capability	Opportunity	Motivation	Physical Capability	Psychological Capability	Physical Opportunity	Social Opportunity	Reflective Motivation	Automatic Motivation	Education	Persuasion	Incentivisation	Coercion	Training	Restriction	Environmental Restructuring	Modelling	Enablement	Communication/Marketing	Guidelines	Fiscal	Regulation	Legislation	Environmental/Social Planning	Service Provision
Armstrong et al., 1999 [46]	✓		✓		✓			✓		✓	✓								✓	✓		✓			
Callahan et al., 2022 [55]	✓				✓					✓	✓							✓	✓	✓					
Daniels et al., 2007 [47]	✓	✓	✓		✓	✓		✓			✓					✓		✓	✓	✓				✓	
DiTosto et al., 2021 [56]	✓	✓	✓		✓	✓	✓		✓	✓	✓							✓	✓	✓		✓			✓
Donaldson et al., 2015 [65]	✓	✓	✓		✓	✓		✓		✓	✓					✓			✓					✓	✓
Dunn et al., 1998 [63]	✓		✓		✓			✓		✓	✓								✓	✓					
Findley et al., 2008 [48]	✓	✓	✓		✓	✓		✓		✓	✓					✓			✓	✓		✓		✓	
Frew et al., 2013 [49]	✓		✓		✓			✓		✓	✓								✓	✓					
Frew et al., 2014 [50]	✓		✓		✓			✓		✓	✓								✓	✓					
Frew et al., 2016 [44]	✓	✓	✓		✓		✓	✓		✓	✓								✓	✓					✓
Kriss et al., 2017 [45]	✓	✓	✓		✓	✓		✓		✓	✓					✓			✓	✓				✓	✓
Larcher et al., 2001 [57]	✓	✓			✓	✓				✓						✓		✓		✓					
Ma et al., 2012 [58]	✓	✓	✓		✓	✓		✓		✓	✓	✓				✓			✓	✓	✓			✓	
Maltezou et al., 2012 [51]	✓				✓					✓										✓					✓
McPhee et al., 2003 [59]	✓	✓	✓	✓	✓		✓	✓		✓	✓					✓		✓	✓	✓		✓		✓	✓
Nicoleau A et al., 2001 [51]	✓		✓		✓			✓		✓	✓								✓	✓					✓
Nyamathi et al., 2015 [60]	✓	✓	✓		✓		✓	✓		✓							✓	✓		✓		✓			
Peterson et al., 2019 [66]		✓	✓	✓			✓	✓		✓	✓	✓				✓		✓		✓	✓			✓	✓
Sagy et al., 2018 [64]		✓					✓			✓									✓						
Schwartz et al., 2006 [53]			✓					✓			✓											✓			
Stringer et al., 2006 [61]		✓	✓		✓			✓		✓										✓					✓
Wood et al., 1998 [54]	✓	✓	✓		✓		✓	✓		✓	✓							✓	✓					✓	✓
Zibrik et al., 2018 [62]	✓	✓	✓		✓		✓	✓		✓								✓	✓	✓				✓	✓
TOTAL (*n* = studies)	19	15	19	2	20	7	8	18	1	21	17	2	0	0	0	8	1	9	17	19	2	6	0	9	11

**Table 4:** Summary of BCTs identified in the vaccine interventions.

BCT Group	BCT	Armstrong et al., 1999 [46]	Callahan et al., 2022 [55]	Daniels et al., 2007 [47]	DiTosto et al., 2021 [56]	Donaldson et al., 2015 [65]	Dunn et al., 1998 [63]	Findley et al., 2008 [48]	Frew et al., 2013 [49]	Frew et al., 2014 [50]	Frew et al., 2016 [44]	Kriss et al., 2017 [45]	Larcher et al., 2001 [57]	Ma et al., 2012 [58]	Maltezou et al., 2012 [51]	McPhee et al., 2003 [59]	Nicoleau A et al., 2001 [51]	Nyamathi et al., 2015 [60]	Peterson et al., 2019 [66]	Sagy et al., 2018 [64]	Schwartz et al., 2006 [53]	Stringer et al., 2006 [61]	Wood et al., 1998 [54]	Zibrik et al., 2018 [62]
1. Goals and planning	1.2. Problem solving																						✓	
	1.3. Goal setting (outcome)																						✓	
2. Feedback and monitoring	2.1. Monitoring of behaviour by others without feedback							✓					✓					✓						
	2.5. Monitoring of outcome(s) of behaviour without feedback																						✓	
3. Social support	3.1. Social support (unspecified)															✓								
	3.2. Social support (practical)							✓								✓		✓	✓				✓	✓
4. Shaping knowledge	4.1. Instruction on how to perform the behaviour	✓					✓	✓								✓		✓	✓					✓
	4.2. Information about Antecedents	✓		✓				✓	✓		✓	✓	✓	✓	✓	✓		✓						✓
5. Natural consequences	5.1. Information about health consequences		✓			✓	✓	✓	✓	✓	✓	✓	✓		✓	✓	✓	✓						✓
	5.2. Salience of consequences						✓		✓	✓														
	5.3. Information about social and environmental consequences													✓										
	5.5. Anticipated regret								✓															
6. Comparison of behaviour	6.1 Demonstration of the behaviour																	✓						
	6.2. Social comparison									✓	✓													
7. Association	7.1. Prompts/cues	✓			✓	✓								✓	✓	✓		✓		✓			✓	
	7.7 Exposure								✓															
8. Repetition and substitution	8.1. Behavioural practice/rehearsal																	✓						
9. Comparison of outcomes	9.1. Credible source			✓	✓	✓		✓			✓	✓		✓		✓	✓	✓	✓		✓			
	9.2. Pros and cons	✓					✓	✓														✓		
	9.3. Comparative imagining of future outcomes								✓															
10. Reward and threat	10.1. Material incentive (behaviour)													✓					✓					
	10.10. Reward (outcome)															✓								
12. Antecedents	12.1. Restructuring the physical environment				✓	✓		✓				✓	✓	✓					✓					
	12.2. Restructuring the social environment															✓								
	12.5. Adding objects to the environment		✓	✓	✓	✓							✓						✓					
13. Identity	13.2. Framing/reframing								✓	✓		✓		✓			✓						✓	✓
No. of BCT within each study		4	2	3	4	5	4	8	7	4	4	5	5	7	3	9	3	9	6	1	1	1	6	5

**Table 5:** Factors influencing vaccination uptake and intervention implementations.

Study	Hesitancy/ Predictors Factors	Barriers to Uptake	Facilitators to Uptake	Intervention Challenges
Callahan et al., 2022 [55]	- Women who routinely got flu shot in the past more likely to get it during pregnancy. - Significant proportion of non-usual flu shot recipients choose to receive it while pregnant. - Smaller proportion who usually got flu shot were uncertain or unwilling to receive it during pregnancy.	- Black race and lower educational attainment associated with lower rate of influenza immunization or plans to be immunized.	- Positive association between respondents’ prior flu shot practices and their opinions about its use in pregnancy. - Previous routine flu shot in the past more likely to get it during pregnancy.	- Interest in counselling and educational materials for the flu shot was low, and practically non-existent among those planning not to be vaccinated during pregnancy. - Small sample size from the Florida site, caused by the early cessation of the study at the onset of the COVID-19 pandemic. - Survey was not formally validated. - Edited version of informational text from the CDC not tested before the study. - Survey conducted at just two clinical sites; thus, findings are not generalizable.
Daniels et al., 2007 [47]	**Reasons for declining:** Fear of shots; need more information; fear of vaccine-related illness; do not believe it is necessary.	- Lack of knowledge, transportation, trust in healthcare system, and culturally relevant information on adult immunizations. - Language and reading level barriers; and fear lack of legal status.	NR	NR
DiTosto et al., 2021 [56]	- Receiving influenza vaccine during pregnancy among the post-guideline cohort was associated with older age, race and ethnicity, initiating prenatal care in the first trimester, and increased number of prenatal visits.	NR	- Each additional prenatal visit associated with increased odds of receiving Tdap vaccine and influenza vaccine.	- Unable to examine whether racial disparities in vaccine uptake widened or were ameliorated after the release of the ACIP 2012 recommendations. - Did not have data on provider recommendations, hence, unable to determine proportion provided a Tdap, influenza, or pneumococcal vaccine recommendations from their provider.
Donaldson et al., 2015 [65]	- **Reasons for declining:** unawareness or never informed about the vaccine; insufficient vaccine information; safety concerns (more research evidence to show efficacy/safety needs); trust in natural immunity and lifestyle, and breastfeeding gives baby enough immunity; whooping cough in childhood has given enough immunity; religious reasons. - **Perceived risks and safety concerns: **side-effects of the vaccine on unborn baby and self.	- Feeling uninformed, lack of professional encouragement and uncertainties of risk and benefit of the vaccine. - Information given difficult to interpret or discuss. - **Low perception of susceptibility reasons:** vaccine considered unnecessary; insufficient risk of contracting the disease; association of personal healthy lifestyle with ‘low risk’ of getting the disease; breastfeeding provided all the immunity needed by babies.	- Encouragement or recommendation from healthcare professional known to them. - Acting in the best interests of unborn baby by protecting themselves and reducing risk of baby developing pertussis in the early weeks following birth. - Identifying with the disease by knowing someone who experienced pertussis. - Personal experience of vaccine-preventable illness.	
Dunn et al., 1998 [63]	NR	- **Reasons for non-completion:** not keeping scheduled 2-month health maintenance visit; no rescheduled appointment during the study period; leaving practice before 2-month health maintenance visit; previous poliovirus vaccine administration to the child; difficulty understanding spoken English; failure to complete the second questionnaire.	NR	- Not giving adequate time to read the VIS before being asked to consent to vaccinations. - Participants cannot read and attend to their children at the same time; feel they know the information since going through similar process before with an older child; and relying on HCW to inform them verbally.
Frew et al., 2013 [49]	NR	NR	- Perceiving illness as very serious and being susceptible to becoming ill with influenza during pregnancy influenced intention to vaccinate babies.	NR
Frew et al., 2014 [50]	NR	NR	- Believe influenza vaccine was >80% effective. - Perceived higher susceptibility of becoming ill with influenza during pregnancy. - Normative support surrounding immunizations increased intent to obtain seasonal influenza vaccine. - Being previously vaccinated.	
Frew et al., 2016 [44]	NR	- Concern about vaccine harm, low perceived influenza infection risk, history of immunization non-receipt.	NR	- Only used single exposure to maternal influenza immunization persuasive messaging.
Kriss et al., 2017 [45]	NR	- No recommendation for Tdap from their doctor; not knowing about Tdap; unsure of Tdap use; did not think they were at risk for tetanus, diphtheria, or pertussis; do not generally take vaccines.	NR	- Less engagement with iBook than video. iBook considered less relatable and more difficult to understand than the video.
Larcher et al., 2001 [57]	NR	- Late bookings and delivery elsewhere (acknowledged problems in inner city areas). - Immunisation in ethnic minority families due to poor understanding of English. - Mobility of families.	NR	- Difficulty in contacting non-attenders with name changes.
Ma et al., 2012 [58]	NR	NR	NR	- Financial constraints access barriers encountered by underinsured and uninsured program participants, limited English proficiency, pastors limited time.
Maltezou et al., 2012 [51]	**Reasons for vaccine refusal: **not wanting the vaccine, self-perception of not being at risk for contacting influenza.	NR	**Significant factors associated with increased vaccination rates**: being of Roma origin or an immigrant, giving birth to neonate with birth weight < 2500 g, and residing in a family with ≥4 family members.	- Operational and financial issues during implementation of cocooning strategy in hospitals. - Involvement of healthcare professionals from different backgrounds (neonatologists, paediatricians, nurses, and obstetricians) traditionally unfamiliar with vaccinations. - Cocooning strategy costly and requires significant human resources for identification and vaccination service provision to all household contacts, while offering several vaccination opportunities, including during evening hours or weekends.
McPhee et al., 2003 [59]	**Predictors for three doses of HepB**: older children, number of years since the parent immigrated, household income above the poverty line, having health insurance.	- Inaccurate knowledge about transmission modes; believe smoking cigarettes or another person’s coughing or sneezing transmitted virus.	- Parents’ marital status; child receiving at least one DTP shot likely to receive three HepB. - Older children in the media campaign area were more likely to receive three HepB.	- Not possible to determine eligibility for about a quarter of eligible participants who were unreachable or refused interview. - Obtaining reports from providers time-consuming and required repeated follow-up calls and re-mailing and/or refaxing the validation forms and accompanying letter. - Some providers required written consent from parents before releasing child vaccination status; and some denied identified child was their patient if no record exist for the child in the county system. - Some information about the child given to providers deemed insufficient to allow accurate identification of the child. - Unable to obtain information from several providers due to insufficient contact information given by parents or because providers never responded. - Few parents could find records reporting vaccination dates.
Nicoleau et al., 2001 [51]	Vaccine not recommended or not recommended strongly; perception of no benefit of taking vaccine; history of sickness after vaccination; too afraid; know people who became ill; egg allergy; never had influenza.	NR	NR	NR
Nyamathi et al., 2015 [60]	**Predictors of vaccine noncompletion**: Asian and Pacific Islander ethnicity; experiencing high levels of hostility; positive social support; history of injection drug use; released early from California prisons; admitted for psychiatric illness.	NR	- **Predictors of vaccine series completion: **reporting six or more friends, recent cocaine use, and staying in drug treatment for at least 90 days.	- Policies enacted in the California state prison system, particularly realignment (or reducing state prison population by transferring inmates to county jails) affected vaccination completion.
Peterson et al., 2019 [66]	Refusal due to perceptions around importance and efficacy. **Factors affecting vaccine uptake, delivery and PHC access:** challenges to navigating the health system; transnational use of health services; language and literacy; expectations of vaccination delivery (comparison of vaccination programmes between countries); vaccine acceptance; vaccine accessibility (appointment booking and appointment length and Vaccination reminders); trust.	NR	NR	- Insurance pilot program where insurance payments would be accepted for vaccinations insured people showed accounting outweighed the benefits, so discontinued.
Schwartz et al., 2006 [53]	History of sickness from past vaccination; knowing someone who got sick; fear of side effects; vaccine will not prevent flu; fear of needles; not wanting it; flu not a serious disease; do not want to get the flu.	NR	NR	- Vaccine supplies exhausted before expected study end date.
Stringer et al., 2006 [61]	There were no differences between the acceptors and non-acceptors with respect to their behavioural and attitudinal HBV beliefs.	NR	- Awareness of vaccination importance during pregnancy. - Time after childbirth engender greater trust of providers to overcome feelings of trust and acceptance of medical recommendations by the providers who helped childbirth experience.	- HBV vaccination program studied not part of usual care and was responsibility of a specific study team; hence, acceptance rates were likely higher vaccination offered as part of the usual care of pregnant adolescents. - No data on completion of entire vaccine series capture; therefore, cohort may not be substantively protected from HBV.
Wood et al., 1998 [54]	NR	NR	- Children receiving private insurance, four or more well-child visits and first-born children. - Mother worked in past year, non-smoker. - Families with fewer life difficulties. - Increased knowledge of the immunization schedule and immunization contraindications.	- Intervention cost high at USD 1587 per child. Overall cost effectiveness compares unfavourably with other medical interventions.
Zibrik et al., 2018 [62]	NR	- Limited knowledge and awareness of HBV vaccination/prevention/treatment; limited English proficiency and eLiteracy skills. - System and provider level barriers to accessing HBV care, and immigration-related barriers. - Inability to travel to appointments due age- or health-related mobility challenges, perceived inconvenience or long distance associated with traveling to a medical appointment. - **HBV Risk Perception: **confident that they do not have HBV because they do not have accompanying symptoms; not having time/too busy to see a doctor; having other health concerns that take priority (e.g., diabetes or cancer); lacking motivation to get tested. - **Awareness and knowledge of HBV: **not aware of risk of infection, transmission route, permanency or severity, prevention and treatment options.; unaware of existence of HBV; uninformed HBV is preventable through vaccination; unaware that immigrant populations from high endemic countries are at high risk for HBV; misconceptions about HBV transmission in communities; stigmatizing and discriminating against HBV carriers. - **Language and eLiteracy Barriers**: challenges finding a doctor who speaks their language; difficulties communicating with their doctor; eLiteracy barriers, such as inability to use a computer or access the internet; challenges assessing the quality of health information online. - **System and Provider Level Barriers**: challenges at the system level to accessing care including limited finance, cost of health services, inability to find a family doctor and how to navigate the healthcare system and locate services; service delays including long waits for testing and medical appointments and difficulty getting in to see a doctor; feeling of having been dismissed by their primary care providers regarding their requests for HBV screening; uncomfortable raising the topic of HBV with their physicians. - **Immigration- and acculturation-related barriers: **resistance to health check-ups; fearful of seeing doctor after so much time has passed without a health check-up; wanting to solve health problems on their own.	NR	- Qualitative nature of study did not allow extrapolation of exact proportion of community members infected with HBV, difficulty in accessing HBV care, or burden from lack of HBV knowledge.

**Table 6:** Recommendations to develop vaccine interventions for racial and ethnic minority populations.

Recommendation	BCT
Include a range of educational resources that are written (e.g., brochures and information sheets), visual (e.g., video) and interactive (e.g., workshops) to increase awareness Multiple message exposure is likely to be more effective than single message exposure.	Instruction on how to perform the behaviour Prompts/cues Adding objects to the environment Information about health consequences
The content of vaccine messages should: • include pros and cons of getting a vaccine and possible future outcomes rather than focusing solely on what might be lost • address reasons for vaccine refusal	Information about Antecedents Information about health consequences Pros and cons Comparative imagining of future outcomes Framing/reframing
Provide culturally specific information and interpretation in the local languages of the ethnic minority groups.	Information about Antecedents Information about health consequences Adding objects to the environment Framing/reframing
Use a combination of public health strategies/campaigns with direct contact and follow-up services available in a timely manner to address any vaccine queries or concerns.	Exposure Problem solving Credible source Information about health consequences
Train peer health educators and credible sources within the community (e.g., religious leaders) to promote vaccine uptake in community settings.	Restructuring the social environment Credible source Social support
Include family and close contacts when providing vaccine services by sharing educational resources and opportunities for dialogue.	Social support
Use community settings (e.g., community clinics, faith, etc.) to inform people about vaccination.	Restructuring the social environment Restructuring the physical environment Information about health consequences
Provide flexible vaccine clinic hours.	Restructuring the physical environment
Include bilingual medical staff in the community.	Restructuring the social environment
Provide vaccines free of charge to remove the cost barrier from patients.	Material incentive
Use problem-solving of barriers (such as identifying financial barriers or problems with transportation), goal setting and provide instructions on how to take action.	Problem solving Goal setting Instruction on how to perform the behaviour
Include encouragement from professionals to address uncertainties about the risk and benefits of vaccines.	Credible source Monitoring of behaviour by others
Monitoring from credible sources to contact families that do not receive vaccinations, and subsequent contact from health visitors to reinforce the importance of vaccine uptake.	Monitoring of behaviour by others

## Data Availability

No new data generated.

## Supplementary Materials

The following supporting information can be downloaded at: https://www.mdpi.com/article/10.3390/vaccines11071259/s1, Supplementary Table S1: BCT Taxonomy (v1): 93 hierarchically-clustered techniques; Supplementary Figure S2: OVID Medline Search Terms; and Supplementary Table S3: EM Vaccine Strategy Studies – Quality Assessment.
