# Supplementary figures and images for: A Systematic Review of Behaviour Change Techniques within Interventions to Increase Vaccine Uptake among Ethnic Minority Populations
Source: Vaccines (Basel). 2023 Jul 19;11(7):1259. doi: 10.3390/vaccines11071259 (PMC10386142; doi:10.3390/vaccines11071259)

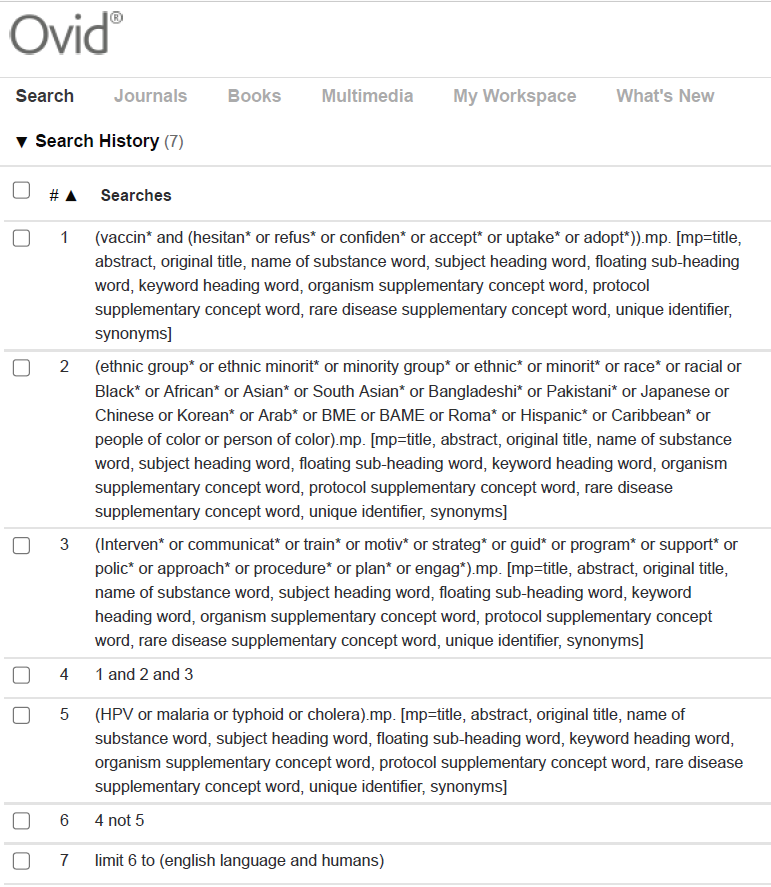

Supplement: Supplementary file 1 [file vaccines-11-01259-s001.zip › Supplementary 2 - OVID Medline Search terms.png]
